# Predictions of the mechanical properties of unidirectional fibre composites by supervised machine learning

**DOI:** 10.1038/s41598-019-50144-w

**Published:** 2019-09-27

**Authors:** M. V. Pathan, S. A. Ponnusami, J. Pathan, R. Pitisongsawat, B. Erice, N. Petrinic, V. L. Tagarielli

**Affiliations:** 1 Engineering Science, University of Oxford, Oxford, OX1 3PJ UK; 2 Mechanical Engineering and Aeronautics, City, University of London, London, EC1V 0HB UK; 3 J P Consultancy Specialists Ltd, Bristol, BS9 3LA UK; 4 Aeronautics, Imperial College London, London, SW7 2AZ UK

**Keywords:** Mechanical engineering, Computational methods

## Abstract

We present an application of data analytics and supervised machine learning to allow accurate predictions of the macroscopic stiffness and yield strength of a unidirectional composite loaded in the transverse plane. Predictions are obtained from the analysis of an image of the material microstructure, as well as knowledge of the constitutive models for fibres and matrix, without performing physically-based calculations. The computational framework is based on evaluating the 2-point correlation function of the images of 1800 microstructures, followed by dimensionality reduction via principal component analysis. Finite element (FE) simulations are performed on 1800 corresponding statistical volume elements (SVEs) representing cylindrical fibres in a continuous matrix, loaded in the transverse plane. A supervised machine learning (ML) exercise is performed, employing a gradient-boosted tree regression model with 10-fold cross-validation strategy. The model obtained is able to accurately predict the homogenized properties of arbitrary microstructures.

## Introduction

The determination of the effective mechanical properties of unidirectional fibre composites from the mechanical properties of their constituents has been widely explored in the past decades. Several analytical approaches exist in the literature to obtain predictions of the effective material constants (for example^1–4^), but these models are approximate and their accuracy is generally low, as shown for example in^5^ for the case of viscoelastic carbon fibre reinforced polymers (CFRPs). For this reason, most authors obtain the macroscopic properties of composites from a multiscale computational homogenisation process, employing the finite element method (FEM) in most cases. This allows establishing relations between microstructural parameters (such as fibre size and volume fraction) and the material’s response, as well as investigating the role of defects, interfaces, and non-linearity in the material behaviour.

High-fidelity FE simulations of the response of unidirectional (UD) composites yield accurate predictions (e.g.^5,6^) but the associated computational time limits their applicability in the design phase. In this study we develop a technique to obtain accurate and computationally inexpensive predictions of the mechanical properties of UD composites as well as to understand their dependence on the microstructural geometry.

The past few years have seen a rapid development of data science and machine learning techniques, and these are increasingly being applied in materials engineering^7–13^. In most of these studies one-to-one relationships between microstructure and material properties are determined. In this study we develop a data-driven supervised machine learning model and apply it to the case of UD fibre composites.

Two-point correlation functions are generally used to decode and quantify the geometry of the material microstructure^14–16^. As detailed representations of such microstructure require a high number of pixels, the size of the 2-point correlation matrix can be quite large. For this reason the image information is typically first compressed using dimensionality reduction techniques such as the principal component analysis (PCA)^17^. Gupta *et al*.^18^ used the principal component regression technique with polynomial basis function to predict the elastic-plastic response of l composite systems considering different inclusion shapes. Cencen *et al*.^19^ recently developed a unified prediction framework using convolutional neural networks (CNN) and the 2-point correlation function, applied to the case of random voxel microstructures. Paulson *et al*.^20^ presented a machine learning homogenization framework for polycrystalline materials. Abuomar *et al*.^10^ used support vector machines to classify vapor-grown carbon nanofibre/vinyl ester nanocomposites in ten different classes, based on their microstructure and mechanical performance.

Applications of such techniques to fibre-reinforced composites are still lacking in the literature. This study presents the development of a data-driven model to predict the elasto-plastic response of a UD fibre composite loaded in its transversely isotropic plane (2–3). We combine PCA and machine learning to link the appearance of composite microstructures to their stiffness and yield strength, at negligible computational cost. The resulting model is driven by data generated by FE simulations and it could be employed in concurrent multi-scale simulations; with suitable modifications, the model could be driven by experimental data.

The paper is organised as follows: Section 2 describes the technique to encode the microstructure geometries, Section 3 presents the details of the computational framework and results are discussed in Section 4.

## Quantitative Description of Composte Microstructures

The analysis presented in this study is based on 2D representations (images) of microstructures, which are sufficient to describe UD composites loaded in the 2–3 plane. The microstructures are statistical volume elements (SVEs), the morphology of which contains random arrays of circular fibres of equal radii, embedded in a uniform matrix. As a prerequisite to the quantification process, the two-phase composite comprising of fibre and matrix shall be digitized into a binary system with 0 and 1 denoting the matrix and the fibre respectively.

A quantitative description of a microstructure should be able to associate a set of measureable parameters to each image, in such a way that two geometrically distinct microstructures are associated to two distinguishable sets of metrics. Performing a simple pixel-by-pixel comparison is not invariant respect to rotation and translation and therefore it is not viable.

A variety of statistical metrics have been used in the literature (e.g.^21,22^) to define the geometry of random composite microstructures comprising non-overlapping fibres in a matrix. This includes metrics such as nearest-neighbour fibre distance and orientation, Ripley’s K function and pair-correlation function, among others. One drawbacks of these metrics is that they do not guarantee a complete and unique characterisation of a microstructure: for example, it is possible to construct two distinct microstructures with the same nearest neighbour orientation function but different Ripley’s K functions.

It has been shown by Niezgoda *et al*.^11,15^ and Torquato^23^ that the *n*-point correlation function provides a rigorous quantification of the geometry of a fibre composite microstructure. In this study, we will focus on the particular case of the 2-point correlation function. A 1-point correlation function defines the cumulative probability of finding a given material phase in the microstructure, i.e. it coincides with the volume fraction of a certain phase. The 2-point correlation function defines the probability of finding phases *p* and *p*′ (here fibre or matrix) at the head and tail, respectively, of a vector of length *r*, randomly placed in the microstructure represented by a square voxel image of size $\sqrt{S}\times \sqrt{S}$ pixels. Each pixel in the image is identified by a unique 2D position vector *s*. The 2-point correlation function is therefore defined as

1 $${f}_{r}^{pp^{\prime} }=\frac{1}{S}\mathop{\sum }\limits_{s=1}^{S}{m}_{s}^{p}{m}_{s+r}^{p^{\prime} }$$

where the microstructure function ${m}_{s}^{p}$ is a function taking value of either 1 (if the material phase *p* is present at the spatial position *s*) or 0 (if any other different phase is located at such spatial position). Note that the function ${f}_{r}^{pp^{\prime} }$ is symmetric when it is an auto-correlation function (i.e. *p* = *p*′) and not symmetric when it is a cross-correlation function (i.e. *p* ≠ *p*′). Note also that the subscript of the microstructure function denotes a spatial position, while the superscript refers to the phase. *S* is the total number of pixels in the image.

It has been shown in^24^ that for the case of a two-phase composite the four 2-point correlation functions ${f}_{r}^{FF},{f}_{r}^{MM},{f}_{r}^{FM}$ and ${f}_{r}^{MF}$ (M and F denote matrix and fibres, respectively) are interrelated, such that only one of the four needs to be calculated; such computation takes time proportional to *S*^2^. In this study, we chose to compute the inter-fibre spatial correlation ${f}_{r}^{FF}$. Fast Fourier transforms (FFTs) have been used in literature to allow for fast computation, by recognizing the convolutional nature of Eq. (1).

**Figure 1:**
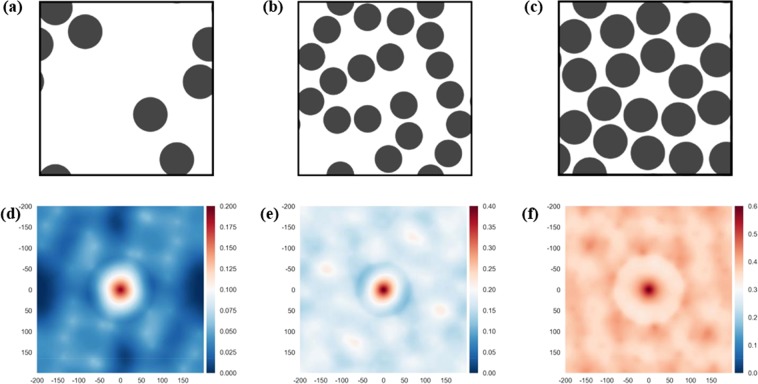
Top: example of SVEs with different fibre volume fractions and radii. Bottom: corresponding contours of the 2-point correlation function *f*_*r*_^*FF*^. (**a**,**d**): ${\varphi }_{f}=0.2;\,{R}_{f}=5\,{\rm{\mu }}m$; (**b**,**e**): ${\varphi }_{f}=0.4;\,{R}_{f}=4\,{\rm{\mu }}m$; (**c**,**f**) ${\varphi }_{f}=0.6;\,{R}_{f}=5\,{\rm{\mu }}m$. The labels on the axes of (**d**–**f**) refer to the number of pixels.

The 2-phase composite microstructures analysed in this study had a total of 160,000 pixels (and 2-point spatial correlations). Examples are shown in Fig. 1a–c; the 1800 microstructures generated had different fibre volume fractions *φ*_*f*_ and fibre diameter *R*_*f*_ (the subscript *f* denotes fibre). A first order quantitative description of the geometry of these microstructures would rely on only two non-dimensional parameters: (i) the volume fraction of one of the phases and (ii) the ratio of fibre radius to size of the SVE analysed. Such simplification is widespread in the literature but does not keep into account some important information on the relative positions of the fibres, the regularity or irregularity of their arrangement, etc. These geometrical details are likely to affect the material’s response and should be taken into account. On the other hand, implementing machine learning exercises considering all 160,000 2-point correlation values would be computationally prohibitive, and most of this information would cause little to no effects on the predicted physical behaviour of the composite.

With these considerations in mind we employ a standard dimensionality reduction technique in data science, the principal component analysis (PCA). The technique projects datapoints from a *n*-dimensional space onto a space of lower dimensions. The axes of such low-dimensional space (principal component axes) are chosen to maximise the variance of the set of projected datapoints population and are numbered by decreasing variance, such that the first principal component (*PC*_1_) is associated to the greatest variance and so on^25^.

Given a dataset of *n* points with *p* dimensions, PCA projects the dataset along *m* orthogonal dimensions (*m* < *p*). In this study, consider a matrix *A*, of order *n* × *p*, containing the 2-point correlation functions for each pixel (*p* = 160000) of the *n* = 1800 microstructures generated. PCA results in an eigen-decomposition of the original dataset *A* of the microstructure ensemble, represented as

2 $$A=(\begin{array}{ccc}f{r}_{n=1,p=1}^{FF} & \cdots  & f{r}_{n=1,p=160000}^{FF}\\ \vdots  & \ddots  & \vdots \\ f{r}_{n=1800,p=1}^{FF} & \cdots  & f{r}_{n=1800,p=160000}^{FF}\end{array})=U\Sigma {V}^{T}$$

where $V=\{{v}_{1},{v}_{2},\ldots ,{v}_{m}\}$ is a matrix of order *p* × *m* containing *m* sets of orthonormal *p* × 1 vectors; these are known as *loadings* and represent the eigenvectors of the symmetric matrix *A*^*T*^*A*, while the corresponding eigenvalues (known as the *principal component variances*) are denoted as $\{{\lambda }_{1},{\lambda }_{2},\ldots ,{\lambda }_{m}\}$. $U=\{{u}_{1},{u}_{2},\ldots ,{u}_{m}\}$ is a matrix of order *n* × *m* containing *m* sets of orthonormal *n* × 1 vectors defined by ${u}_{i}\equiv \frac{1}{\sqrt{{\lambda }_{i}}}{v}_{i}$. The positive real terms ${\sigma }_{i}\equiv \sqrt{{\lambda }_{i}}$ are called *singular values*. $\sum$ is a diagonal matrix of order *m* × *m* containing the singular values *σ*_*i*_. The principal components are ranked by sorting their corresponding singular values *σ*_*i*_, each of which represents the fraction of variance of the dataset captured by each individual principal component, in order of decreasing magnitude. In other words, the first principal component has the singular value of the greatest magnitude.

In this study we choose *m* = 50, reducing our 160,000-dimensional datapoints to an equal number of 50-dimensional datapoints. The 50 principal components (PCs) for each point are determined for each microstructure after PCA of the ensemble. The PCs quantify the geometry of the microstructures and are used as input for a machine learning exercise, which provided the mechanical properties as outputs. The computational procedure is illustrated in the following section.

## Computational Procedure

We proceed to describe the details of the proposed computational procedure. The steps performed were as follows:

- 1800 microstructures were generated;
- 2-point correlations functions were evaluated for each microstructure;
- Data dimensionality reduction was performed by PCA; 50 principal components (PCs) were associated to each microstructure;
- FE simulations were performed to simulate the response of each of the 1800 microstructures to 3 loading cases, extracting 5 mechanical properties;
- Machine learning was used to perform a regression, having as input 50 PCs representing the geometry of the microstructure, and as an output the set of 5 mechanical properties above;
- Additional microstructures were generated (regular arrays of fibres) to test the accuracy of the model.

In the following we provide further details on all steps performed.

### Generation of the microstructures

In this study, we consider computer-generated microstructures of UD fibre composites. These are generated using an algorithm previously developed by the authors^21^. This algorithm has been shown to generate realistic and truly random microstructures, which is crucial to ensure exhaustive sampling of the microstructure configuration space, leading to an effective and comprehensive training dataset for the machine learning problem. The fibre distribution was periodic at the edges of the SVEs.

Square SVEs of side length 50 µm were generated, considering six different fibre volume fractions *ϕ*_*f*_ in the range of 10–60% and three different fibre radii, *R*_*f*_ = 4, 5 and 6 µm. For each pair of fibre volume fraction and fibre radius, we generated 100 independent realizations, resulting in a total of 1800 SVEs. The geometry of each realization is saved using a resolution of 400 by 400 pixels, as it was found that this resolution was sufficient to approximate the desired volume fractions within a 3% error margin; this was evaluated in an iterative process. Each microstructural image forms the raw input ${X}_{i}\in \bar{X}$, where *i* = 1,2...1800.

### Two-point correlation function

Evaluation of this function was via (Eq. (1)), placing the origin of the reference system at the centre of the image. Computation of the 2-point correlation function (CF) for all 1800 microstructures required 10 minutes on a machine with 16 CPUs (Intel i7 core of speed 3.2 GHz, with 16 GB of RAM and a solid state hard drive). Examples of three microstructures and their corresponding 2-point correlation ${f}_{r}^{FF}$ are shown in Fig. 1. We note that the value of the CF at the centre of the image represents the fibre volume fraction in each microstructure, and its value is a maximum in the image.

### Dimensionality reduction

**Figure 2:**
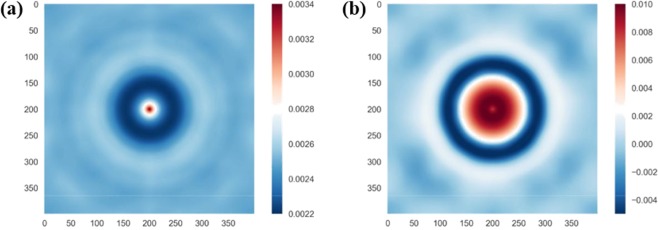
Contours of the relative loadings of each pixel in the (**a**) 1^st^ principal component; and (**b**) 2^nd^ principal component. The labels on the axes refer to the number of pixels.

The 160000-dimensional raw datasets *X*_*i*_ were compressed by performing PCA as described in Section 2. This allowed to compute 50-dimensional dataset, ${X}_{\bar{i}}=\{P{C}_{1}^{i},P{C}_{2}^{i}\ldots P{C}_{50}^{i}\}$ to be used as an input by the machine learning model. These inputs were sorted in order of their relative importance (or maximum variance). The relative loadings (i.e. relative weight of each pixel) of the first two principal components are shown in Fig. 2. For the first principal component, the highest weight is given to the centre of the 2-point correlation image, carrying information on the fibre volume fraction. The second principal component takes into account the second most dominant geometric feature of the microstructure, the diameter of the fibres.

**Figure 3:**
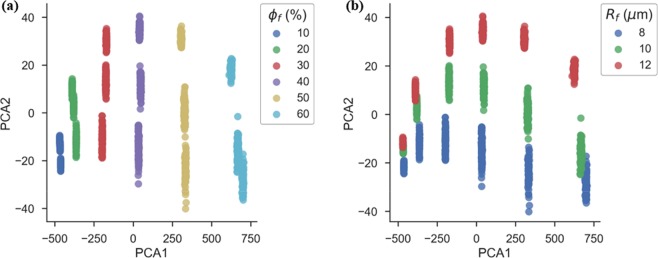
Score plot of the first two principal components, for all the 1800 microstructures generated.

The plot of scores of each data point, i.e. the location of each datapoint in the new reduced principal component space are shown in Fig. 3. As discussed above and evident from the figure, the first principal component predominantly carries information of the most dominant geometric feature, the fibre volume fraction. The second principal component tracks the second dominant feature, the fibre radius. Similar interpretation of principal components of higher order is not straightforward, however they must encode other geometric features such as interfibre distance, fibre clustering, periodicity, etc.

### Finite element analysis

For each microstructure in the input set *X*_*i*_ we determined the corresponding homogenized, macroscopic mechanical properties *Y*_*i*_ using FE simulations in the commercial FE solver Abaqus Standard. The fibres were modelled as an isotropic, linear elastic solid with properties similar to those of glass fibres (Young’s modulus *E*_*f*_ = 30 GPa, Poisson’s ratio *v*_*f*_ = 0.2). The matrix was modelled as an isotropic, elastic-perfectly plastic solid (incompressible *J2* plasticity) with Young’s modulus *E*_*m*_ = 3 GPa Poisson’s ratio *v*_*m*_ = 0.3 and yield strength *σ*_*yield*_ = 120 MPa, representative of an epoxy resin. The size of the domain (50 µm) was chosen based on analyses presented in^5,6,21^.

**Figure 4:**
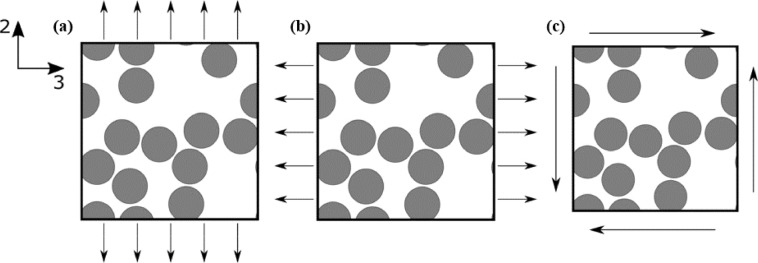
Schematics of applied loading conditions on the SVE: (**a**) uniaxial tension in direction 2, (**b**) uniaxial tension in direction 3, and (**c**) pure shear in the 2–3 plane.

The 2-D geometry was discretized via the 4-noded plane strain quadrilateral CPE4 elements of Abaqus, following a mesh convergence study. Periodic boundary conditions were imposed on opposite sides of the SVEs and 3 quasi-static loading cases were considered: uniaxial tension in directions 2 and 3, as well as pure shear, as shown in Fig. 4. Normal or shear strains up to 4% were imposed by prescribing the displacements of appropriate dummy nodes; this was chosen to be sufficiently large to ensure an elastic-plastic transition in all cases.

The simulations predicted an initial elastic response transitioning to an elasto-plastic response with increasing applied strains. The stress versus strain histories were analysed to extract the homogenized transverse Young’s moduli *E*_22_ and *E*_33_, the transverse shear modulus *G*_23_, the transverse normal yield strengths *σ*_22_ and*σ*_33_. The yield strengths were evaluated as the flow stress at plastic normal or shear strains of 0.2%. The yield strength in shear *σ*_23_ was not extracted in all cases, due to difficulties in convergence of the FE simulations, and therefore it is not included in the machine learning exercise. The output dataset is therefore ${Y}^{i}=\{{E}_{22}^{i},{E}_{33}^{i},{G}_{23}^{i},{\sigma }_{22}^{i},{\sigma }_{33}^{i}\}$. Note that the moduli and strengths in directions 22 and 33 are expected to be similar, due to the random isotropic nature of the microstructure.

### Machine learning application

This multi-output regression problem was treated as five independent single-output problems. To avoid overfitting we use the standard shuffled, 10-fold cross-validation technique to get an unbiased estimate of the model performance. This was implemented in Python language using a scikit-learn library (ver 0.20.0) and the associated “GradientBoostingRegressor” algorithm.

The set of inputs ${X}_{\bar{i}}=\{P{C}_{1}^{i},P{C}_{2}^{i}\ldots P{C}_{50}^{i}\}$ and outputs ${Y}^{i}=\{{E}_{22}^{i},{E}_{33}^{i},{G}_{23}^{i},{\sigma }_{22}^{i},{\sigma }_{33}^{i}\}$ for each of the 1800 microstructures was divided in 10 randomly populated subsets, containing data for 180 microstructures each. 90% of the data in these subsets (representing 162 microstructures) was used for training purposes, while the remaining 10% (18 microstructures) were used for validation. The accuracy of all regressions was measured by *r*^2^, defined as

$${r}^{2}(y,\hat{y})=1-\,\frac{{\sum }_{i=0}^{{n}_{samples}-1}{({y}_{i}-{\hat{y}}_{i})}^{2}}{{\sum }_{i=0}^{{n}_{samples}-1}{({y}_{i}-\bar{y})}^{2}}$$

where *y* are the set of training values, $\hat{y}$ are the set of predicted values obtained using a machine learning algorithm, and $\bar{y}=(\mathop{\sum }\limits_{i=0}^{{n}_{samples}-1}{y}_{i})/{n}_{samples}$.

In a preliminary study we evaluated multiple regression models of varying complexities: a linear regression with a 2^nd^ order polynomial basis function, a 3^rd^ order polynomial regression and tree-based algorithms such as random forests. It was found that the Gradient-Boosted tree Regressor (GBR) model provides the most accurate predictions, with the highest mean *r*^2^ metric for all the five outputs. GBR is an ensemble technique that relies on sequentially fitting new decision trees to the residual of prior decision trees. The GBR model depends on 3 parameters to be selected by the user: learning rate, number of estimators and maximum depth of individual decision trees. To select the optimal values of these 3 parameters we used a nested grid-search optimization approach in conjunction with the 10-fold cross-validation strategy to avoid data-leakage, i.e. overfitting of the model. The procedure was as follows:

- The search space for the 3 parameters to use with the GBR was discretised;
- For each choice of the 3 parameters, the GBR was used on each of the 10 subsets of data, and the average *r*^2^ obtained in all 10 subsets was calculated, and used as a measure of accuracy;
- The optimal values of the 3 parameters were determined (these were: learning rate = 0.1; number of estimators = 800; max depth of decision trees = 3)
- The GBR was trained one final time using the optimal set of paterameters and the entire dataset (comprising data for all 1800 microstructures); again, 90% of the data was used for training and the remaining 10% was used for validation.

A complete description of the GBR model and associated parameters is out of the scope of this present work, hence the reader is referred to^26^ for further information.

## Results and Discussion

**Figure 5:**
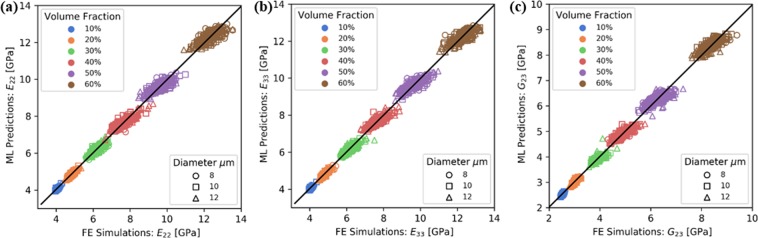
Comparisons of the predictions of FE simulations and those of the proposed machine learning approach, for the elastic properties of the material.

**Figure 6:**
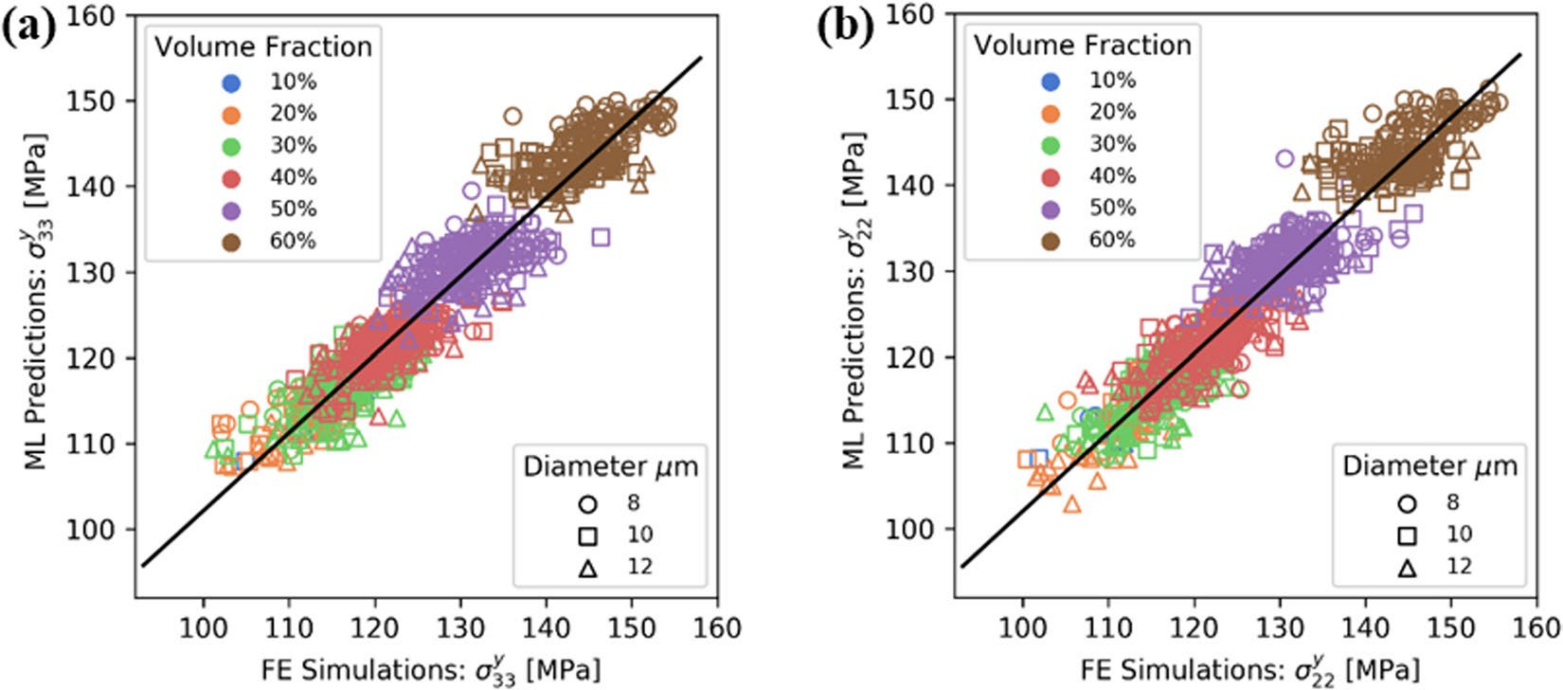
Comparisons of the predictions of FE simulations and those of the proposed machine learning approach, for the transverse yield strengths of the composite.

Figures 5 and 6 present FE predictions for all 1800 microstructures, compared to the data-driven cross-validation predictions of an optimal GBR model. For both elastic properties (Fig. 5) and yield strengths (Fig. 6) the machine learning model shows a margin of error of at most 5%. This is satisfactory in consideration of the small size of the training dataset examined in this study. The accuracy can be improved by increasing the number of training datapoints and avoiding the discretisation of the ranges of volume fractions and fibre radius explored, but rather sampling the parameters randomly within these ranges.

**Figure 7:**
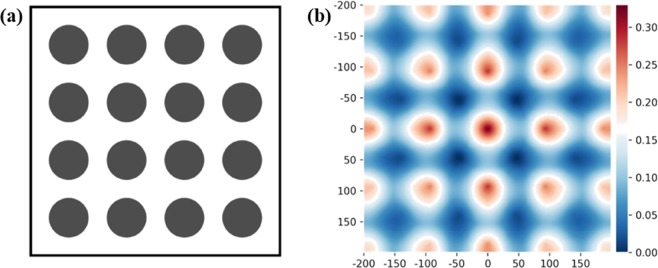
(**a)** Example of a square periodic microstructure with 16 fibres $({\varphi }_{f}=0.33)$; (**b**) Contours of the corresponding 2-point correlation function; the labels on the axes refer to the number of pixels.

As a further validation exercise we use the machine learning model to predict the homogenised properties of microstructures with periodic fibre distributions. We performed additional FE simulations for 3 different square periodic RVEs, containing square arrays of 4, 9 and 16 fibres resulting in volume fractions *ϕ*_*f*_ of 8%, 18% and 33%, respectively. An example of a microstructure containing 16 fibres and the corresponding 2-point correlation function is shown in Fig. 7.

The machine learning predictions were compared with the results of detailed FE calculations; in Table 1 we present the results of this comparison: clearly, the model’s predictions are quite accurate. The accuracy is similar to that shown in Figs 5 and 6, while these regular arrays of fibres have profound geometric differences with the random arrays of fibres used in the training phase, as it is also evident from the appearance of the contours of the 2-point correlation functions shown in Fig. 7b, compared to those shown in Fig. 1. This suggests our algorithm has acquired, by training, a form of primordial “understanding” of what geometric parameters set the macroscopic values of the mechanical properties of a UD fibre composite. We note that predictions are less accurate for the microstructure with 16 fibres; this is due to the fact that such microstructure has a larger volume fraction, with reduced distance between the fibres, which makes the predictions more challenging.

**Table 1:** Percent difference between predictions of the machine learning algorithm and results of FE simulations, for the case of microstructures with square periodic arrays.

Number of fibres	*E*_22_	*E*_33_	*G*_23_	${{\boldsymbol{\sigma }}}_{{\bf{22}}}^{{\boldsymbol{y}}}$	${{\boldsymbol{\sigma }}}_{{\bf{33}}}^{{\boldsymbol{y}}}$
4	2.43 %	0.68 %	0.23 %	0.17 %	1.32 %
9	2.44 %	3.44 %	2.99 %	0.30 %	3.33 %
16	3.96 %	4.46 %	6.7 %	1.06 %	3.18 %

The results of the proposed regression depend of the choice of the number of PCs used to represent the geometry of the microstructure. To illustrate such dependence in Table 2 we show, for the case of the transverse modulus *E*_22_, the training running time and the validation accuracy as a function of the number of PCs used. Clearly an increase of such number to more than 10 increases the training running time but does not affect notably the accuracy of the predictions. A similar trend is observed for the other macroscopic mechanical properties, with the difference that for the case of *G*_23_ and the two yield strengths considered the accuracy tends to be lower (as evident from Fig. 6). The choice of the first 50 PCs in this study is a compromise between accuracy and runtime, together with the fact that using a relatively large number of inputs (50 rather than, say, 10) makes the machine learning exercise performed more challenging.

**Table 2:** Sensitivity of the training running time and validation accuracy to the number of PCs used, for the case of the transverse modulus *E*_22_.

Number of PCs	Training Runtime (s)	Mean *r*^2^
10	81	99.1
20	139	99.44
50	338	99.45
70	493	99.47
100	697	99.48
200	1,155	99.5
400	1,183	99.5

The effectiveness of the machine learning exercise depends obviously on the size of the training dataset. To illustrate the sensitivity of the accuracy of the predictions to the number of microstructures used for training, in Table 3 we report the *r*^2^ score (average across the 10 subsets of the 10-fold cross validation strategy) to the size of the training set, for the cases of predictions of *E*_22_ and *σ*_22_. The accuracy initially increases steeply with the training set size, but such increase becomes less rapid when more than 720 microstructures are used.

**Table 3:** Sensitivity of the *r*^2^ score (average across the 10 subsets of the 10-fold cross validation) to the size of the training set, for predictions of *E*_22_ and *σ*_22_.

Size of training set	*r*^2^ for predictions of *E*_22_	*r*^2^ for predictions of *σ*_22_
180	0.576512	0.265424
360	0.566154	0.427627
540	0.713846	0.634068
720	0.763077	0.707797
900	0.836923	0.752034
1080	0.886154	0.76678
1260	0.910769	0.796271
1440	0.923077	0.840508
1620	0.947692	0.855254
1800	0.964265	0.873654

**Figure 8:**
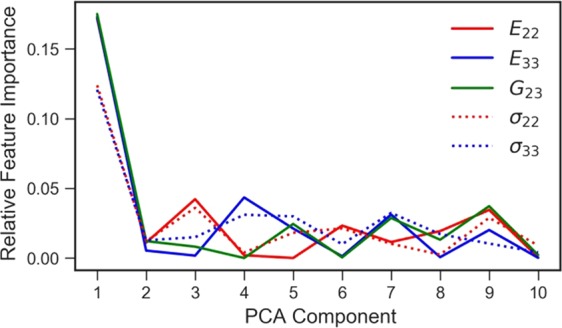
Influence of principal components on predicted properties.

The regression method employed in this study allows evaluating the relative weights of each principal component in the calculation of the outputs, extracting the most dominant features from independent decision trees used in the gradient-boosted regressor algorithm. This information is shown in Fig. 8 for the first 10 PCs. As expected, the dominant feature influencing the predictions is the fibre volume fraction, encoded by the 1st principal component. We note that the yield stresses are less sensitive to fibre volume fraction than the elastic moduli, as it can be inferred from comparing Figs 5 and 6. The PCs of higher order have less weight in the predictions, however these weights are non-negligible.

**Figure 9:**
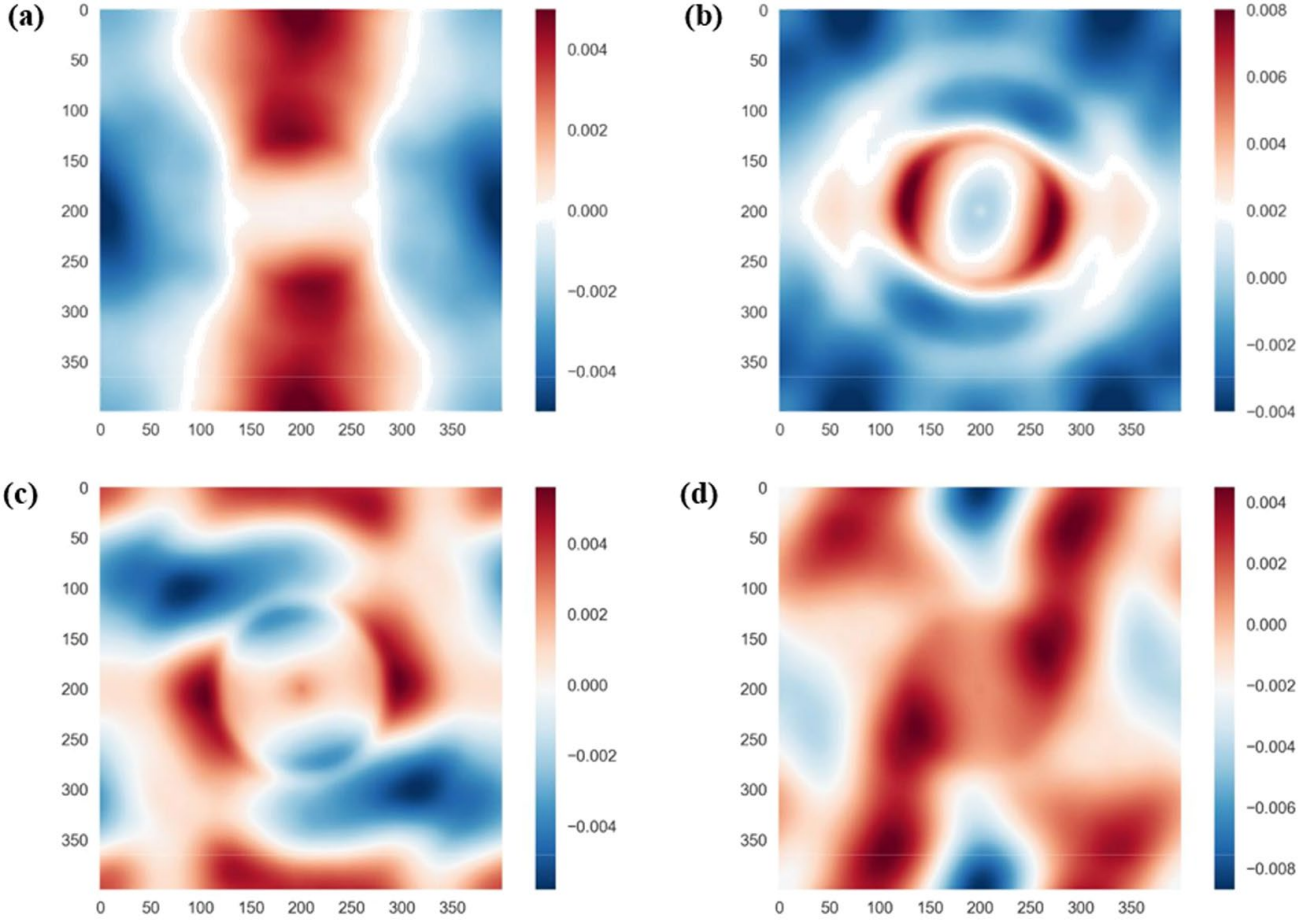
Relative loadings of selected principal components. (**a**) 3rd principal component; (**b**) 4th principal component; (**c**) 7th principal component; (**d**) 9th principal component.

We finally show in Fig. 9 the relative loadings of each pixel in the 2-point correlation function, for selected principal components of high importance (as deduced from Fig. 9). The 3rd principal component (Fig. 9a), which is an important regression feature for *E*_22_ and σ_22_, is seen to primarily take into account the arrangement of fibres in direction 2 (vertical). Similarly, the 4th principal component (Fig. 9b) has a large weight in determining *E*_33_ and carries information regarding the arrangement of fibres in direction 3. The 7^th^ PC (Fig. 9c) is important for predictions of *E*_33_ and *G*_23_. Interestingly, the 9^th^ PC (Fig. 9d) has a relatively high importance for predictions of all the material properties considered in this study.

The proposed modelling methodology allows savings in computational costs which depend on the computational efforts required by the FE simulations. In the case discussed in this paper the FE models are relatively light in computational terms, such that predictions of the 5 mechanical properties considered takes approximately 30 minutes; the same predictions are performed via machine learning in approximately 0.5 s, 3600 times faster. Such saving in computational time can be several orders of magnitude greater for complex material models, involving millions of degrees of freedom; in such cases the FE analyses would be much longer to run, while our proposed strategy would still yield fast predictions of the mechanical properties after appropriate training.

## Conclusions

The 2-point correlation function and its principal component analysis are used to quantify the geometry of the microstructures of UD fibre composites. The first 50 PCs are used as an input in a machine learning exercise employing a gradient-boosted tree regression model. The training data for such exercise are provided by the predictions of detailed FE simulations, providing the non-linear material response. This results in an inexpensive computational procedure able to predict the macroscopic elastic stiffness and yield strengths of the UD composites, starting from an image of the composite’s microstructure. Training of the machine learning model is performed using a 10-fold, cross-validation strategy utilising 1800 FE simulations, and the resulting predictions have accuracy of order 5%; the model also allows ranking the effects of different geometric features on the macroscopic mechanical properties of the composites, showing that the properties investigated are dominated by the fibre volume fraction.

In this study we focus our attention on the macroscopic elastic moduli or yield strengths, however the method can be extended to predictions of the full elasto-plastic material response. The fast predictions of this approach could be used to inform multiscale constitutive models. With some modifications, similar approaches could be devised to be driven by experimental data, i.e. from micrographs of the microstructure and actual force measurements during mechanical testing, rather than from computer-generated microstructures and the corresponding FE analyses. In the case of fibre composites, similar approaches can be devised to explore data-driven predictions of the response in the case of fibre-dominated failure modes. We leave these as topics for future studies.
